# Severe Transverse Myelitis Following Ankle Surgery Potentially Unmasking Late-Onset Systemic Lupus Erythematosus

**DOI:** 10.7759/cureus.74006

**Published:** 2024-11-19

**Authors:** Harrison Jordan, Sidharth Sahni, Matthew Nguyen, Michael Moradi, Shiraz Mumtaz, Leonard Johnson

**Affiliations:** 1 Physical Medicine and Rehabilitation, ECU Health, Greenville, USA; 2 Physical Medicine and Rehabilitation, New York University, New York, USA; 3 Anesthesiology, Drexel University College of Medicine, Philadelphia, USA; 4 Physical Medicine and Rehabilitation, Drexel University College of Medicine, Philadelphia, USA; 5 Orthopedics, Drexel University College of Medicine, Philadelphia, USA; 6 Internal Medicine, ECU Health, Greenville, USA

**Keywords:** acute transverse myelitis (atm), autoimmune neuro, autoimmune neurology, lupus myelitis, sytemic lupus erythematosus

## Abstract

A 70-year-old female underwent open reduction and internal fixation (ORIF) of her right ankle following a mechanical trip and fell two weeks before hospital admission. Two weeks following surgery, the patient experienced sudden-onset bilateral anterior thigh paresthesias and burning mid-back pain. Over the ensuing two days, the patient developed bilateral lower extremity weakness, bilateral lower extremity numbness, and urinary retention with constipation, which led to hospital presentation. A non-contrast cervical/thoracic/lumbar spine MRI on the day of admission revealed a possible syrinx from T3-T12. A repeat thoracic spine MRI revealed enhancement of the spinal cord at T9-T10 and T11-T12, suggestive of transverse myelitis or spinal cord infarction. Initiation of IV methylprednisolone sodium succinate improved strength and sensation, and IVIG (intravenous immunoglobulin) therapy was initiated. Transverse myelitis is typically idiopathic or attributed to infectious causes or systemic autoimmune conditions. Spinal cord injury remained high on the differential diagnosis, considering transverse myelitis rarely presents in the postoperative period. While methylprednisolone sodium succinate is the first-line treatment for transverse myelitis, its role remains unclear in treating spinal cord injury. Given the patient's positive response to methylprednisolone sodium succinate, an autoimmune panel was sent to determine the underlying etiology, resulting in a positive ANA (antinuclear antibody) and anti-dsDNA. Thus, transverse myelitis may be an initial presentation of systemic lupus erythematosus (SLE). In rare cases of spinal cord injury versus immune-mediated disorders affecting the spinal cord, corticosteroid treatment should be considered pending diagnosis confirmation.

## Introduction

Transverse myelitis is an uncommon condition that typically occurs in the second or fourth decade of life and has an annual incidence that ranges from 1.34 to 4.60 cases per million [1,2]. It is typically idiopathic or attributed to infectious causes, systemic autoimmune conditions, or central nervous system disease [3]. We present an unusual case, lupus myelitis, as the initial presentation of systemic lupus erythematosus (SLE). This presentation developed into severe myelitis, resulting in the inability of the patient to ambulate and lower limb paresthesia. It is important for physicians to consider corticosteroid treatment in rare cases of spinal cord injury versus immune-mediated disorders affecting the spinal cord.

## Case presentation

A 70-year-old female with a past medical history (PMH) of hyperlipidemia presented with sudden-onset bilateral anterior thigh paresthesias followed by burning mid-back pain. The patient had a mechanical fall two weeks prior in which she fractured her right ankle. This led to an open reduction and internal fixation (ORIF) of her right ankle, and she was discharged without any complications. Two weeks after surgery, the patient experienced sudden-onset bilateral anterior thigh paresthesias, subsequently accompanied by burning mid-back pain. Over the next few days, the lower extremity weakness and numbness progressively worsened to the point where she could not stand from a chair, and she experienced urinary retention and constipation, which prompted a hospital visit.

**Figure 1:**
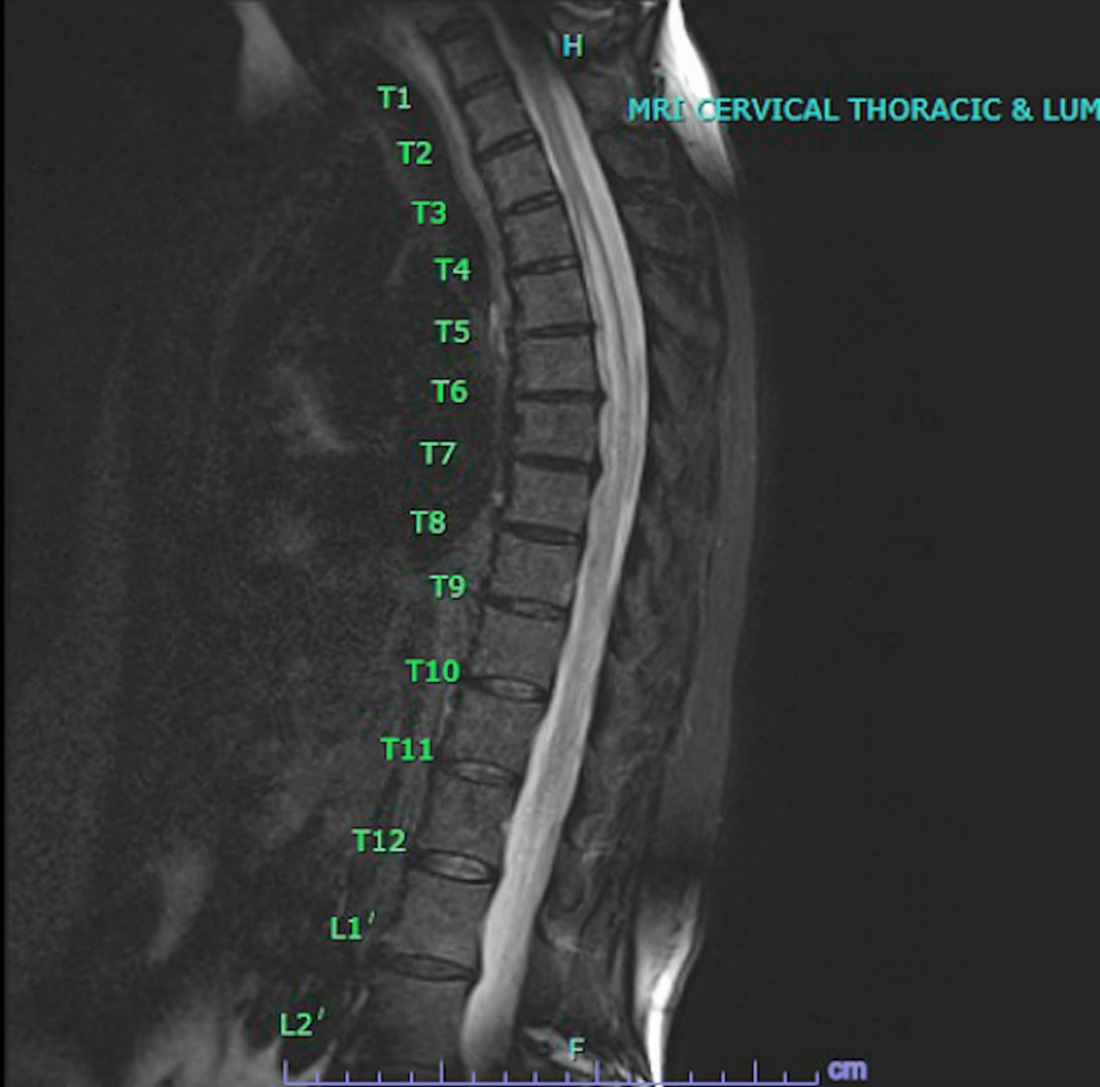
MRI cervical/thoracic/lumbar spine without contrast showed possible syrinx from T3-T12

The neurologic assessment unveiled profound bilateral lower extremity weakness, characterized by 1/5 strength in hip and knee flexion and extension, as well as ankle dorsiflexion and plantar flexion, albeit evaluation of the latter was hindered by a splint encasing the right ankle. The sensory examination revealed a notable reduction in sensation and a concurrent presence of numbness extending bilaterally in her lower extremities. Upon palpation from the umbilicus to the knee, allodynia was noted. Noteworthy preservation of toe proprioception was observed bilaterally. Neurological reflexes exhibited a 3+ response in patellar and Achilles reflexes bilaterally, except for the right Achilles reflex, which was not evaluable. On admission to the hospital, a non-contrast cervical/thoracic/lumbar spine MRI revealed a possible syrinx from T3-T12 (Figure 1).

**Figure 2:**
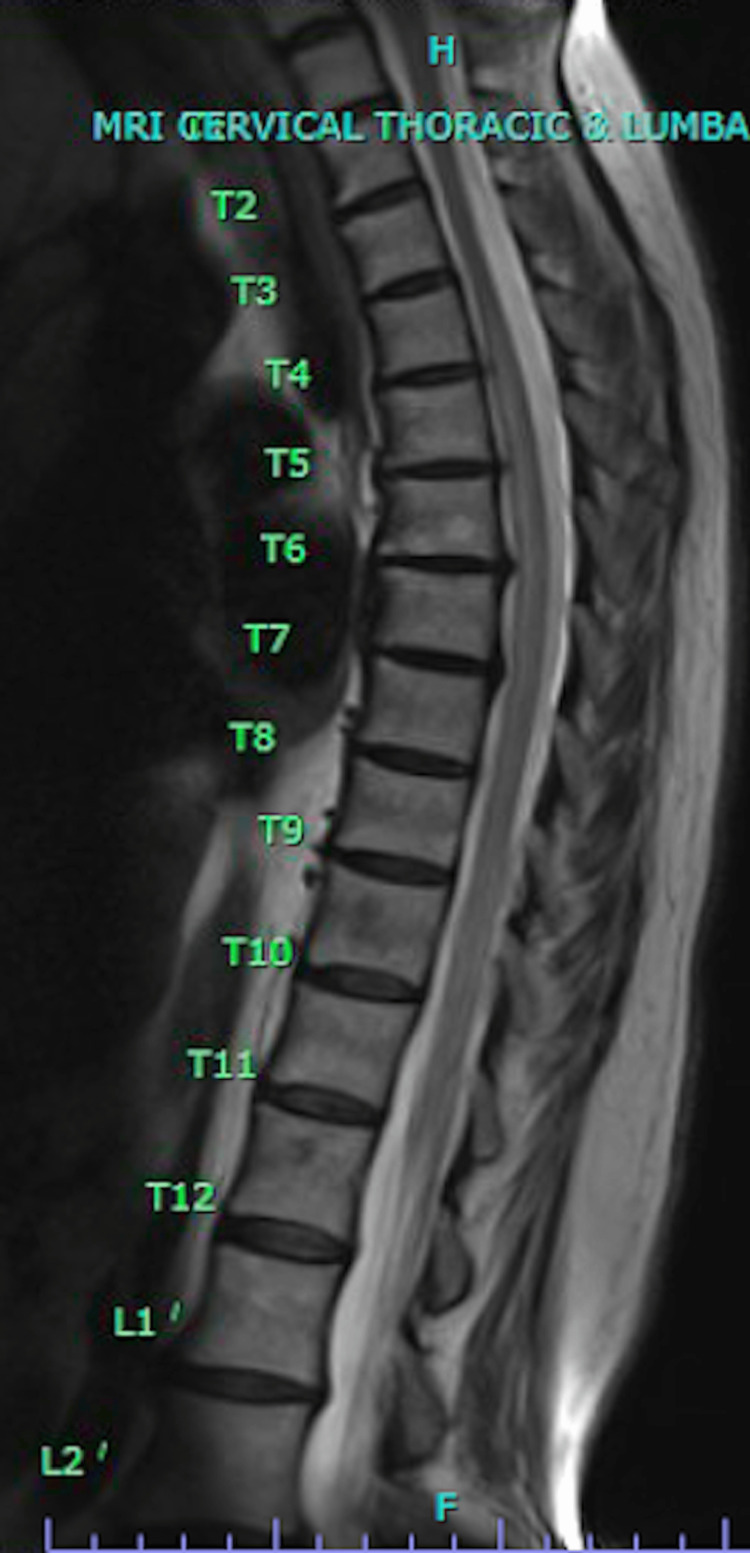
MRI of the T-spine without contrast reveals enhancement of the spinal cord from T9-T10 and T11-T12, suggestive of spinal cord infarct or transverse myelitis

A repeat thoracic spine MRI revealed enhancement of the spinal cord at T9-T10 and T11-T12, suggesting transverse myelitis (TM) or spinal cord infarction (Figure 2). Neurology recommended initiating IV methylprednisolone sodium succinate (MPSS) followed by IVIG. Neurosurgery was consulted, and it was believed that a spinal angiogram was low yield and that an AV (arteriovenous) fistula was unlikely. After receiving MPSS, the patient's strength and sensation improved, and the patient completed a course of IVIG. While completing the course of IVIG, the patient was found to be positive for anti-dsDNA antibodies, suggesting a diagnosis of lupus myelitis. Upon discharge from the hospital, the patient regained complete strength and was scheduled to follow up with rheumatology.

## Discussion

TM is an uncommon condition that typically occurs in the second or fourth decade of life and has an incidence of only one to eight cases per million per year [4]. It is typically idiopathic or attributed to infectious causes, systemic autoimmune conditions, or central nervous system disease [4]. When presented with TM in the setting of SLE, the diagnosis is lupus myelitis, which is a rare condition that occurs in 0.5%-1% of patients with SLE. In those patients, 30%-60% may present with TM as the presenting feature [5,6].

Moreover, a lack of history of autoimmune disease seen in this patient with no history of SLE symptoms makes this presentation atypical, especially following surgery. Spinal cord infarction remained high on the differential diagnosis, considering TM rarely presents in the postoperative period [6,7]. While MPSS is the first-line treatment for TM, its role remains unclear in treating spinal cord injury [8,9]. Given the patient's positive response to MPSS, an autoimmune panel was sent to determine the underlying etiology, resulting in a positive ANA and anti-dsDNA. Thus, TM may be an initial presentation of SLE. Given these results and presentation, the patient was eventually diagnosed with lupus myelitis.

## Conclusions

In rare cases of spinal cord injury versus immune-mediated disorders affecting the spinal cord, corticosteroid treatment with IV methylprednisolone should be strongly considered pending diagnosis confirmation. Although there is a lack of large studies and a limited number of patients, it is important for physicians to consider prompt treatment with corticosteroids to improve overall prognosis.
